# Tissue banking training courses: Polish experience

**DOI:** 10.1007/s10561-012-9294-4

**Published:** 2012-02-09

**Authors:** Artur Kaminski, Grzegorz Gut, Izabela Uhrynowska-Tyszkiewicz, Ewa Olender

**Affiliations:** 1 Department of Transplantology and Central Tissue Bank, Medical University of Warsaw, ul. Chalubinskiego 5, 02-004 Warsaw, Poland; 2 National Centre of Tissue and Cell Banking, Warsaw, Poland

**Keywords:** Tissue banking, Training courses for medical professionals

## Abstract

Personnel directly involved in the donation, procurement, testing, processing, preservation, storage and distribution of human tissues and cells should be appropriately qualified and provided with timely and relevant training according to EU directives. In the time of new tissue and cells regulations implementation such a training system existed in Poland only at a local level. The first training programme outlines for various groups of health professionals engaged in tissue banking practice was created in co-operation with the Institute for LifeLong Learning at University of Barcelona in 2006. This initial training courses were financially supported by EU Transition Facility Programme 2004. Then, starting from 2006, based on previous experience, system of advanced training courses was created. This training programme was financially supported by the National Programme for the Development of Transplantation Medicine 2006–2009—POLGRAFT financed by Polish Ministry of Health. During 2006 and 2007 first set of tissue banking initial training courses were provided according to TF 2004 project. Over 200 pathologists, forensic medicine specialists and other medical doctors responsible for donor screening and classification, medical directors of tissue establishments, technical staff; tissue graft users: orthopaedic surgeons, neurosurgeons, cardiosurgeons and ophthalmologists were trained. Between 2006 and 2009 there were organized 8 advanced tissue banking training courses according to POLGRAFT programme. There were organized both theoretical and practical courses on various aspects of tissue for over 350 persons. We present our experience in organisation of international and national tissue banking training courses.

## Introduction

Tissue banking is a highly specialized procedure that requires relevant professional qualifications of personnel involved in the donation, procurement, testing, preparation, preservation, storage and distribution of human tissue grafts. This is one of the key elements that affect the safety and quality of tissue grafts used for clinical applications. Directive 2004/23/EC of the European Parliament and the Council of 31 March 2004 on setting standards of quality and safety for the donation, procurement, testing, processing, preservation, storage and distribution of human tissues and cells (2004) and Commission Directive 2006/86/EC of 24 October 2006 implementing Directive 2004/23/EC of the European Parliament and the Council as regards traceability requirements, notification of serious adverse reactions and events and certain technical requirements for the coding, processing, preservation, storage and distribution of tissue and human cells (2006) provides requirement that personnel of tissue bank must receive basic/initial training and updated training as required when there is a need for changes in procedures or scientific knowledge develops and adequate opportunities for relevant professional development.

At the time of implementation of new regulations governing tissue and cell banking in Poland, such a training system existed only locally. Personnel of other musculoskeletal tissue banks was trained in the Department of Transplantology and Central Tissue Bank of the Medical University of Warsaw, which is the oldest tissue bank in Poland, founded in 1963. Two eye tissue banks were founded in co-operation with Tissue Banking International, and their staff was trained by foreign experts. Another created tissue banks drew knowledge from experience of other already existing banks in Poland and adopted standards of EATB (European Association of Tissue Banks) or EEBA (European Eye Banking Association). Some of personnel was also trained in tissue banks abroad. Transposition of European directives that regulates tissue banking activities into the Polish law in The Act of July 1st, 2005 on cell, tissue and organs recovery, storage and transplantation (2005) obligated the newly created National Centre for Tissue and Cell Banking to organize initial training and continuous education system for medical professionals engaged in tissue banking practices. In accordance with the legal requirements in Poland, tissue bank personnel is required to participate in trainings at least once every 2 years.

National Centre for Tissue and Cell Banking was established in January 2004 by the Polish Minister of Health using the expertise, manpower and infrastructure of the Department of Transplantology and Central Tissue Bank at the Medical University of Warsaw. By 2004 there was not created a system of education of pathologists, forensic specialists, medical doctors and others responsible for the testing of donors and tissue procurement, medical directors of tissue banks and responsible persons, technical staff and medical doctors responsible for tissue graft clinical application. There were already accessible tissue banking distance learning courses based on the e-learning modules organized by the University of Barcelona in Spain (Páez et al. 2003), University of Singapore in co-operation with the International Atomic Energy Agency (Nather et al. 2003) and training programme on a frame of European Public Health Project—European Quality system for Tissue Banking—EQSTB (Kaminski et al. 2007) dedicated to tissue banking staff. All of these training courses were provided only in English language.

## Materials and methods

First cycle of training courses for Polish medical practitioners engaged in tissue banking activities were organized in co-operation with a team of Institute of LifeLong Learning, University of Barcelona as a part of a Transition Facility project 2004/016-829.01.05 entitled Establishment of institutional control on the safety and quality of human tissues and cells used for transplantation—Development of a National Centre for Tissue and Cell Banking.

Before starting of preparation of training courses the education needs were evaluated. In 2004 there were 10 tissue banks operating in Poland: 4 multi-tissue banks and 6 mono-tissue banks (2 heart valves banks, 3 eye tissue banks and 1 skin bank) cooperating with over 200 hospitals. In connection with the enactment of a new European law on tissue and cell banking and the necessity of its introduction in all Member States of the European Union, all asked pointed on the need to discuss this subject during training. Various groups involved in tissue banking at different stages from donor evaluation and tissue procurement through processing and storage to distribution and clinical application pointed at various aspects of these activities, depending on which part of it they were responsible for. Each of the created training courses, based on this evaluation emphasized on different subjects. Therefore, four different types of training courses facing at doctors involved in donor screening and tissue procurement, medical directors of tissue banks and responsible persons, technical staff of tissue banks and medical doctors responsible for clinical application of human tissue grafts were prepared and organized.

This initial series of four training courses was carried out in Warsaw in 2006 and 2007. Due to the participation of foreign lecturers invited by the University of Barcelona, the training was carried out in English language with simultaneous translation. Each course ended with a test of knowledge of its participants, and evaluation of course faculty.

Another programme, independent from the Transition Facility 2004 dedicated to the training of medical personnel involved in the tissue has been introduced in Poland in 2006. This programme was a part of the Public Health Programme—National Programme for the Development of Transplantation Medicine 2006–2009—POLGRAFT financed by the Polish Ministry of Health. This programme was in the assumption of a continuation of the initial training cycle and included several advanced training courses, each of which was dedicated to different aspects of tissue banking. Since all invited speakers came from Poland, this series of trainings was carried out in Polish language only.

## Results

In the initial training organized under the Transition Facility 2004 project in collaboration with the University of Barcelona, attended by 50 pathologists, forensic medicine specialists and other medical doctors responsible for screening of donors and procurement of tissues. The training was conducted in two rounds in December 2006. These two identical 2-day training (16 h) focused on the ethical and legal aspects of transplantation, donor selection criteria, the diagnosis of infectious diseases, tissue specific eligibility criteria, techniques of tissue procurement and the requirements for tissue procurement organization.

Further training for 20 medical directors of tissue banks and responsible persons was organized in March 2007. During this 10-day course (80 h) were discussed the following issues: ethical and legal aspects of transplantology, organization and structure of tissue bank, technical and sanitary requirements for tissue bank, the documentation in the tissue bank, anatomy and histology of human tissue grafts, emerging infectious diseases, criteria for donor selection, standard operating procedures in the tissue bank, a quality management system in tissue bank, tissue grafts sterilization techniques, traceability and vigilance in tissue banking, the procedure for accreditation of tissue banks, and clinical application of tissue grafts.

Training for technical staff of tissue banks has been designed for 5 days (40 h) and included the following topics: ethical and legal aspects of transplantology, the documentation in tissue bank, anatomy and histology of human tissue grafts, donor selection criteria, standard operating procedures in tissue bank, the quality system in tissue bank, traceability of tissue grafts and clinical application of tissue grafts. The course was organized twice in March and April in 2007 and was intended for 50 persons.

The last of this cycle of an initial courses was organized in June 2007. The course was organized for 80 doctors including orthopedic surgeons, ophthalmologists and cardiac surgeons. During the first day of a training a general issues were discussed including: ethical and legal aspects of transplantology, donor selection criteria and documentation required for tissue graft application procedure. During the second day of clinical training indications for application of human tissue grafts were are discussed in the subgroups, depending on the specialty.

Each of the organized courses was evaluated by participants both in terms of content and organization of more than 4.5 in a 1–5 scale of assessments (where 1 meant the weakest note and 5—the best).

As part of the National Programme for the Development of Transplantation Medicine 2006–2009—POLGRAFT between 2006 and 2009, eight training courses were organized. Three 2-day courses were organized in September 2006 for 80 persons, in October 2008 for 50 people, and in September 2009 for 70 participants. These courses were aimed to transplant coordinators, directors of tissue banks and responsible persons and for tissue procurement organizations. Courses were devoted to cooperation and coordination of tissue donation, criteria for donor selection, tissue procurement procedures, conditions for transportation of tissues procured, analysis and risk assessment in tissue banking, adverse reactions and events associated with tissue banking.

Four other 3-day training courses were organized for the banks directors and responsible persons. In November 2006 training for 20 persons was dedicated to organization and maintenance of clean rooms in tissue banks. Two additional workshops were organized in September and November of 2007. First one, attended by 36 persons was devoted to good manufacturing practice and its application in tissue banking. Second, organized for 10 attendees was dedicated to public relations in tissue banking. Another in this series of training was organized in November 2008. The subject of this course on good manufacturing practice and quality assurance was attended by 50 persons.

In October and November of 2009 a series of theoretical and practical training for working in cleanrooms of tissue banks was organized in Research and Training Tissues and Cells Bank of the National Centre for Tissue and Cell Banking. Training was held for 21 directors of tissue banks and responsible persons, and for 17 participants recruited from tissue bank technical staff. The first course consisted of theoretical part and practical simulation of work in cleanroom of tissue bank. The course was divided into two identical trainings consisted of a one-day theoretical and a one-day practical part. The practical part was held in the premises of tissue bank. This training was organized in tissue bank during the technical break of the operation when the premises were not used for processing of tissue grafts. After the training conducted in tissue bank facilities planned replacement of HEPA filters and other maintenance procedures (yearly servicing and technical regulations of the systems) were done. Then, before the start of the routine work of the tissue bank, cleaning procedures and qualification of cleanrooms (microbiology and particle count) were performed.

Feedback information from participants attending courses provided under National Public Health Programme was very positive. However, this series of trainings was not officially evaluated regarding its organisation and content as it was done for the initial trainings organized under the Transition Facility 2004 project.

All participants of the training courses received course certificates. These certificates of training in accordance with legal requirements in Poland, are one of the elements evaluated at each inspection carried out in tissue bank. These inspections are held no less frequently than once every 2 years. Above all, however, certificates from training courses are very important when a tissue bank every 5 years is applying to the Minister of Health for authorization of its activites.

## Conclusions

Due to our experience based on organization of training courses in Poland during the years 2006–2009 for more than 550 tissue banks staff members and physicians responsible for application of human tissue grafts, trainings should be designed differently in different groups of health professionals involved in the activities of tissue banking. Practical training in cleanrooms of tissue banks can be organized only for a small group of participants and never during routine work in tissue bank.

Presence of larger group of trainees in cleanrooms may create a problem in tissue bank due to an increased risk of contamination and loss of air classes in cleanrooms. On the other hand, in our opinion, training in a real cleanroom is better than simulated training. Introducing simulation training should, however, precede any practical training of persons with no prior experience in working in cleanrooms of tissue banks. Our experience in creating a training system for employees of the tissue may be helpful in developing training programmes for other institutions.
